# Gait Analysis of Conventional Total Knee Arthroplasty and Bicruciate Stabilized Total Knee Arthroplasty Using a Triaxial Accelerometer

**DOI:** 10.1155/2016/6875821

**Published:** 2016-08-25

**Authors:** Takenori Tomite, Hidetomo Saito, Toshiaki Aizawa, Hiroaki Kijima, Naohisa Miyakoshi, Yoichi Shimada

**Affiliations:** ^1^Kitaakita Municipal Hospital, 16-29 Shimosugiaza Kamishimizusawa, Kitaakita City, Akita 018-4221, Japan; ^2^Department of Orthopedic Surgery, Akita University Graduate School of Medicine, Hondou 1-1-1, Akita City, Akita 010-8543, Japan

## Abstract

One component of conventional total knee arthroplasty is removal of the anterior cruciate ligament, and the knee after total knee arthroplasty has been said to be a knee with anterior cruciate ligament dysfunction. Bicruciate stabilized total knee arthroplasty is believed to reproduce anterior cruciate ligament function in the implant and provide anterior stability. Conventional total knee arthroplasty was performed on the right knee and bicruciate stabilized total knee arthroplasty was performed on the left knee in the same patient, and a triaxial accelerometer was fitted to both knees after surgery. Gait analysis was then performed and is reported here. The subject was a 78-year-old woman who underwent conventional total knee arthroplasty on her right knee and bicruciate stabilized total knee arthroplasty on her left knee. On the femoral side with bicruciate stabilized total knee arthroplasty, compared to conventional total knee arthroplasty, there was little acceleration in the *x*-axis direction (anteroposterior direction) in the early swing phase. Bicruciate stabilized total knee arthroplasty may be able to replace anterior cruciate ligament function due to the structure of the implant and proper anteroposterior positioning.

## 1. Introduction

Conventional total knee arthroplasty (TKA) can include preservation of the posterior cruciate ligament (PCL) (cruciate-retaining, CR), removal of the PCL (posterior stabilized, PS), and substitution of the PCL (cruciate-substituting or cruciate-sacrificing, CS), but the anterior cruciate ligament (ACL) is still removed, and the knee after TKA has ACL dysfunction. Therefore, there are cases that experience paradoxical motion, in which the femur exhibits anterior slipping in early flexion; this is considered one of the causes of poor results after TKA [1].

Victor and Bellemans developed bicruciate stabilized (BCS) TKA to solve this problem [2]. BCS TKA is believed to reproduce ACL function in the implant and provide anterior stability. Changing the shape of the articulating surfaces and the thickness of the polyethylene reportedly causes medial pivot motion and roll back close to that of a normal knee joint [3].

Conventional TKA on the right knee and BCS TKA on the left knee were performed in the same patient. A triaxial accelerometer was then fitted to both knees after surgery, and gait analysis was performed.

## 2. Case Presentation

**Figure 1:**
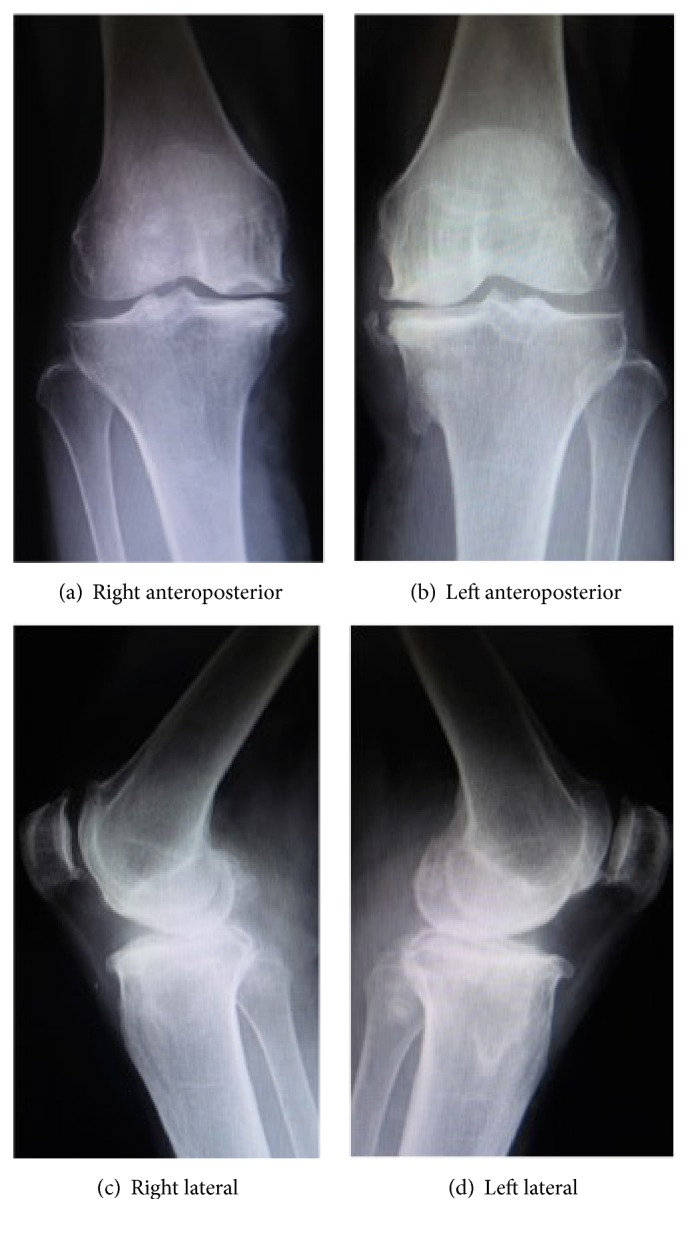
Preoperative X-ray.

A 78-year-old woman had experienced pain in both knees since around 2005. Conservative medical treatment at a nearby clinic failed to mitigate her symptoms, and she was referred to our hospital in 2012. Preoperative range of motion was −10° and 130° in right knee extension and flexion, respectively, and −5° and 130° in left knee extension and flexion, respectively. The preoperative X-ray showed equivalent deformation on the left and right (Figure 1).

In 2012, she underwent right TKA, for which the implant was the Scorpio NRG (Stryker, Mahwah, NJ, USA). It was performed with a medial parapatellar incision, followed by PS and cement fixation, without patellar resurfacing. In 2015, she underwent left TKA, for which the implant was the Journey II (Smith and Nephew, Memphis, TN, USA). That procedure was also performed with a medial parapatellar incision, followed by BCS, cement fixation, and patellar resurfacing.

**Figure 2:**
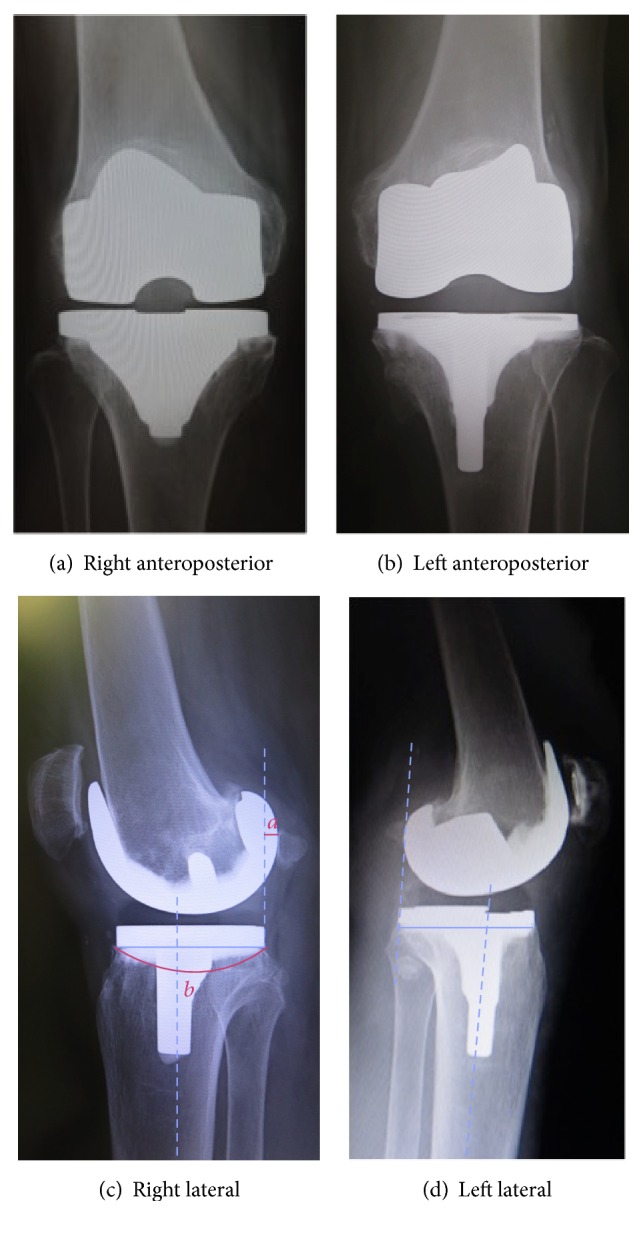
Postoperative X-ray.

Postoperative lateral X-ray images of the extended position showed that the posterior offset ratio (POR) was 12.1% with conventional TKA and 0% with BCS TKA. This POR is the POR (*a*/*b* × 100%) calculated with the knee joint in the extended position, reported by Onodera et al. (Figure 2) [4]. The POR of a normal knee is reportedly 5.63%  ±  5.34%, and BCS TKA is believed to yield anteroposterior positioning that is close to that of a normal knee.

The range of motion of the knee joint was 5° to 130° for the right knee and 0° to 145° for the left knee at three months after the BCS TKA. The new Knee Society Score (2011 KSS) was used for postoperative assessment, yielding equivalent results for the left and right knees (Table 1). Objective knee indicators show higher points in left knee, because the range of motion is better in left knee than right knee. And expectation also shows higher points in left knee, because left knee has better pain relief than right knee. In terms of activity, points of walking on an uneven surface and climbing up or down a flight or stairs are fewer in left knee than right knee.

**Figure 3:**
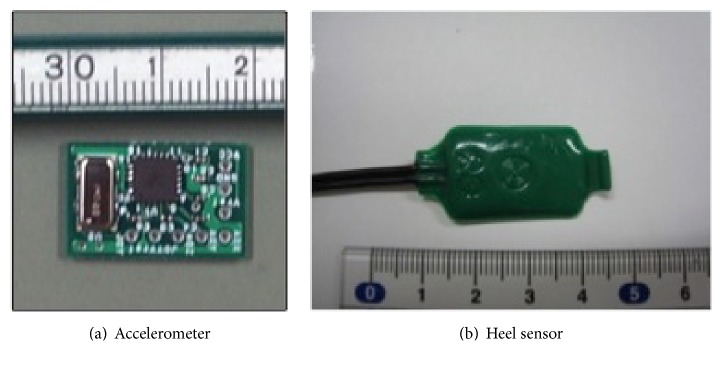
Sensors.

After surgery, the patient was asked to walk with a triaxial accelerometer (Hitachi H48C 3-Axis Accelerometer Module, Hitachi Metals Co., Ltd., Tokyo, Japan) (Figure 3(a)) placed on the upper end of the patella on the femoral side and on the tibial tubercle on the tibial side and a heel sensor (Click BP, Tokyo Sensor Co., Ltd., Tokyo, Japan) (Figure 3(b)) placed on the heel in order to determine the stance phase and the swing phase. For gait conditions, the patient was instructed to walk at normal speed and to walk a flat straight path without using a walking aid.

**Figure 4:**
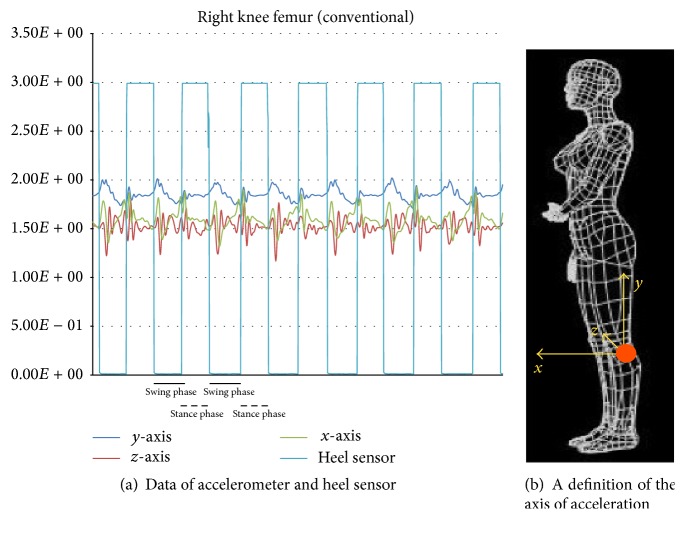
Data of sensors and a definition of the axis.

The resulting data were collected into a data logger (Memory HiLogger LR8431, Hioki E. E. Co., Nagano, Japan). A graph of the actual walking data is presented (Figure 4(a)). The horizontal axis is time, and the vertical axis is voltage (volts (V)). The straight line shown in the graph is the signal of the heel sensor, making it possible to determine the stance phase and swing phase. The accelerometer has an output power of 1.5 V when stopped; in a resting state, 1.5 V is 0 g (gravity) and 1.833 V is 1 g (gravity) (1 g = 9.81 m/s^2^). Acceleration can be interpreted as being positive or negative with reference to 1.5 V. The definition of each acceleration axis is that the *x*-axis is the anteroposterior direction, the *y*-axis in the superoinferior direction, and the *z*-axis is the horizontal direction (Figure 4(b)).

**Figure 5:**
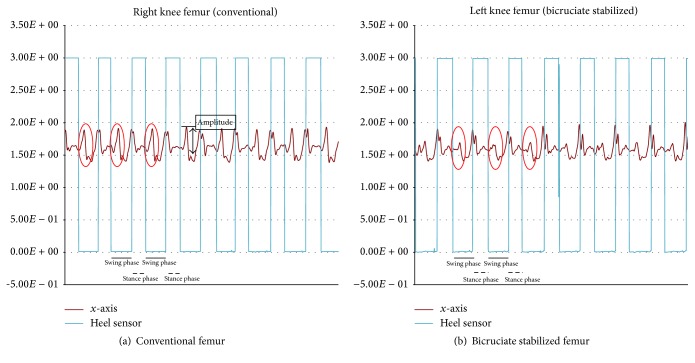
Acceleration of the femur.

**Figure 6:**
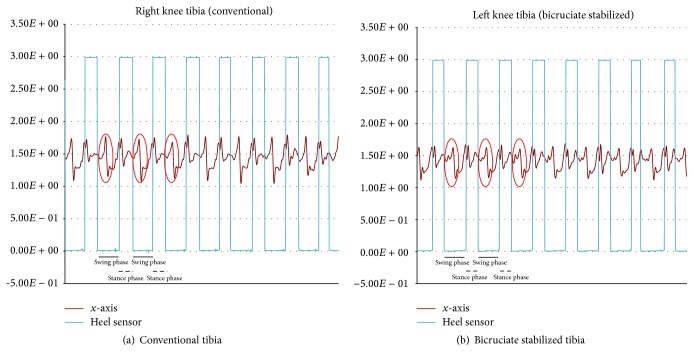
Acceleration of the tibia.

**Figure 7:**
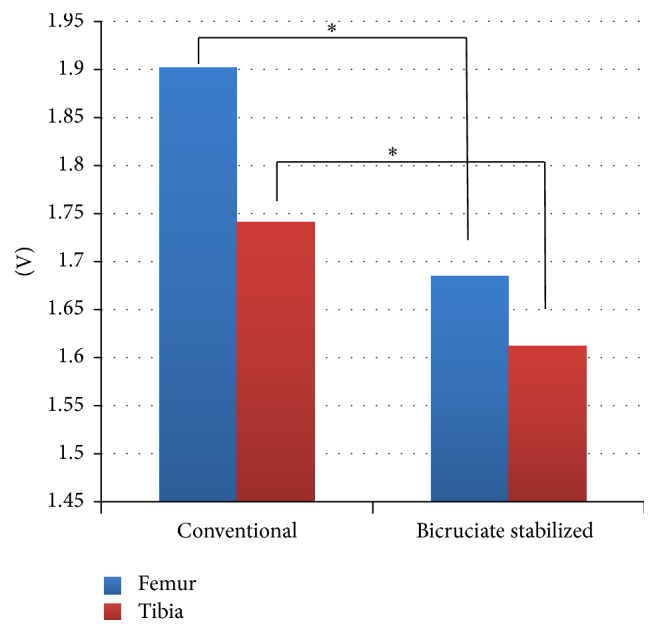
Acceleration of the conventional and bicruciate stabilized total knee arthroplasty. ^*∗*^ *P* < 0.05 paired *t*-test.

Results from walking while wearing the accelerometers on the femur and tibia of the conventional TKA and BCS TKA legs showed a difference in the *x*-axis of the anteroposterior direction, which is presented in more detailed graphs (Figures 5 and 6). The femur of conventional TKA was found to have a greater amplitude of voltage than the femur of BCS TKA. A similar trend was observed on the tibial side, though not as great as the femoral side. When the maximum amplitude is measured with 1.5 V, which is the output power when stopped, as the baseline, then the femoral side and tibial side for conventional TKA were 1.902 V and 1.741 V, respectively, while the femoral side and tibial side for BCS TKA were 1.685 V and 1.612 V, respectively. A left/right comparison showed that, on the femoral side with BCS TKA, compared to conventional TKA, there was little acceleration in the *x*-axis direction (anteroposterior direction) in the early swing phase (*P* < 0.05 paired *t*-test). A similar trend was also observed on the tibial side (Figure 7). All analyses were performed using SPSS ver. 23.0 (IBM Corp., Armonk, NY, USA).

## 3. Discussion

BCS TKA has been said to provide anterior stability, but few reports have quantitatively assessed stability. In this report, accelerometers were used to quantitatively assess anterior stability with left/right comparisons made between conventional TKA and BCS TKA in the same patient.

There have been some reports on motion analysis of the knee using accelerometers, and they are reportedly effective tools for motion analysis [5–7].

Staab et al. used accelerometers and gyroscopes to conduct gait analysis in OA patients and reported that these sensors were approximately the same as the Vicon [5]. Khan et al. conducted gait analysis with accelerometers in a TKA group and a control group, and they reported that the TKA group showed greater acceleration changes than the control group in step-down and turning motions [6]. Liikavainio et al. reported that skin-mounted accelerometers above and below knee had good repeatability in healthy young men [7].

The ACL is said to act as a stabilizer in the early flexion phase [8]. In this study, the analysis confirmed that, in the early swing phase (early flexed phase), there was less acceleration in the anteroposterior direction on the femoral side with BCS TKA than with conventional TKA. This suggests that, with BCS TKA, the knee joint was stabilized in the anteroposterior direction in the early flexion phase, reducing the so-called paradoxical motion said to occur in the early flexion phase with conventional TKA, where the femur exhibits anterior slipping.

## 4. Conclusion

BCS TKA may be able to replace ACL function due to the structure of the implant and proper anteroposterior positioning.

## Figures and Tables

**Table 1:** New knee society score.

	Right conventional	Left bicruciate stabilized
Indicators	63	66
Symptoms	25	25
Satisfaction	36	36
Expectations	8	10
Activities	50	48
